# Identifying residual transmission of lymphatic filariasis after mass drug administration: Comparing school-based versus community-based surveillance - American Samoa, 2016

**DOI:** 10.1371/journal.pntd.0006583

**Published:** 2018-07-16

**Authors:** Meru Sheel, Sarah Sheridan, Katherine Gass, Kimberly Won, Saipale Fuimaono, Martyn Kirk, Amor Gonzales, Shannon M. Hedtke, Patricia M. Graves, Colleen L. Lau

**Affiliations:** 1 National Centre for Epidemiology and Population Health, Australian National University, Acton, Australian Capital Territory, Australia; 2 National Centre for Immunisation Research and Surveillance, Westmead, New South Wales, Australia; 3 Department of Global Health, Research School of Population Health, Australian National University, Acton, Australian Capital Territory, Australia; 4 Neglected Tropical Disease Support Center, The Task Force for Global Health, Decatur, Georgia, United States of America; 5 Centers for Disease Control and Prevention, Division of Parasitic Diseases and Malaria, Atlanta, Georgia, United States of America; 6 American Samoa Department of Health, Pago Pago, American Samoa; 7 Lyndon B Johnson Tropical Medical Center, Pago Pago, American Samoa; 8 Department of Animal, Plant and Soil Sciences, La Trobe University, Bundoora, Victoria, Australia; 9 College of Public Health, Medical and Veterinary Sciences, James Cook University, Cairns, Queensland, Australia; RTI International, UNITED REPUBLIC OF TANZANIA

## Abstract

**Introduction:**

Under the Global Programme to Eliminate Lymphatic Filariasis (LF), American Samoa conducted seven rounds of mass drug administration (MDA) from 2000–2006. The World Health Organization recommends systematic post-MDA surveillance using Transmission Assessment Surveys (TAS) for epidemiological assessment of recent LF transmission. We compared the effectiveness of two survey designs for post-MDA surveillance: a school-based survey of children aged 6–7 years, and a community-based survey targeting people aged ≥8 years.

**Methods:**

In 2016, we conducted a systematic school-based TAS in all elementary schools (N = 29) and a cluster survey in 28 villages on the two main islands of American Samoa. We collected information on demographics and risk factors for infection using electronic questionnaires, and recorded geo-locations of schools and households. Blood samples were collected to test for circulating filarial antigen (CFA) using the Alere Filariasis Test Strip. For those who tested positive, we prepared slides for microscopic examination of microfilaria and provided treatment. Descriptive statistics were performed for questionnaire variables. Data were weighted and adjusted to account for sampling design and sex for both surveys, and for age in the community survey.

**Results:**

The school-based TAS (n = 1143) identified nine antigen-positive children and found an overall adjusted CFA prevalence of 0.7% (95% CI: 0.3–1.8). Of the nine positive children, we identified one microfilariaemic 7-year-old child. The community-based survey (n = 2507, 711 households) identified 102 antigen-positive people, and estimated an overall adjusted CFA prevalence of 6.2% (95% CI: 4.5–8.6). Adjusted village-level prevalence ranged from 0–47.1%. CFA prevalence increased with age and was higher in males. Of 86 antigen-positive community members from whom slides were prepared, 22 (25.6%) were microfilaraemic. School-based TAS had limited sensitivity (range 0–23.8%) and negative predictive value (range 25–83.3%) but had high specificity (range 83.3–100%) and positive predictive value (range 0–100%) for identifying villages with ongoing transmission.

**Conclusions:**

American Samoa failed the school-based TAS in 2016, and the community-based survey identified higher than expected numbers of antigen-positive people. School-based TAS was logistically simpler and enabled sampling of a larger proportion of the target population, but the results did not provide a good indication of the overall CFA prevalence in older age groups and was not sensitive at identifying foci of ongoing transmission. The community-based survey, although operationally more challenging, identified antigen-positive individuals of all ages, and foci of high antigen prevalence. Both surveys confirmed recrudescence of LF transmission.

## Author summary

Lymphatic filariasis (LF) is caused by infection with filarial worms that are transmitted by mosquito bites. Globally, 68 million are infected, with ~36 million people disfigured and disabled by complications such as severe swelling of the legs (elephantiasis) or scrotum (hydrocele). The Global Programme to Eliminate LF (GPELF) aims to interrupt disease transmission through mass drug administration (MDA), and to control illness and suffering in affected persons by 2020. The World Health Organization recommends conducting Transmission Assessment Surveys (TAS) in school children aged 6–7 years, to determine if infection rates have dropped to levels where disease transmission is no longer sustainable. American Samoa made significant progress towards eliminating LF. Following seven rounds of MDA, American Samoa passed TAS in 2011–2012 and 2015, with antigen prevalence of <1%. Despite passing TAS, recent studies have provided evidence of ongoing disease transmission in American Samoa, questioning the suitability of TAS for conducting surveillance after MDA has stopped. We compared a school-based survey of children aged 6–7 years and a community-based survey targeting people aged ≥8 years as tools for conducting post-MDA surveillance of LF. Our study provides recommendations for strengthening of post-MDA surveillance as countries approach the GPELF elimination targets.

## Introduction

Lymphatic filariasis (LF) is a neglected tropical disease caused by *Wuchereria bancrofti* and *Brugia* species of helminth worms. The disease is transmitted by mosquito vectors including *Aedes*, *Anopheles*, *Culex* and *Mansonia* species. Globally, an estimated 68 million people are infected; with 36 million microfilaemic people and 36 million people who are disabled or disfigured because of complications including lymphoedema, elephantiasis and scrotal hydrocoeles [1]. In 2000, the World Health Organization (WHO) launched the Global Programme to Eliminate Lymphatic Filariasis (GPELF), which aims to eliminate LF as a public health problem by 2020. The GPELF includes two strategies, (i) to interrupt transmission of LF by conducting mass drug administration (MDA) in all disease endemic regions, and (ii) morbidity management and disability prevention for infected people [2]. The GPELF is estimated to have delivered 6.2 billion treatments to over 820 million people since its inception [3]. Prior to the formation of the GPELF, the Pacific Programme to Eliminate LF (PacELF) was formed in 1999 to support 22 Pacific Island Countries and Territories in the Western Pacific Region [4]. As of 2017, Cook Islands, Niue, the Marshall Islands, Tonga and Vanuatu have successfully achieved elimination targets established by WHO [5].

WHO recommends conducting Transmission Assessment Surveys (TAS) in children aged 6–7 years for epidemiological assessment of transmission after the completion of MDA [2]. A minimum of two TAS are recommended at 2–3 year intervals, until the absence of transmission can be validated. The first TAS is designed to be conducted at least six months after the final round of MDA to decide if MDA can be stopped, while subsequent TAS are conducted to establish the absence of ongoing transmission. The rationale for choosing children aged 6–7 years as the target population for TAS is because they were born during or after MDA, and any infection in this population would most likely indicate recent and/or ongoing transmission. Transmission is considered not sustainable when the mean antigen (Ag) prevalence in an evaluation unit drops below the TAS threshold. Critical cut-off values are thresholds below which transmission is considered not sustainable and depend on the filarial parasite and vector. Critical cut-off values are calculated so that the likelihood of an evaluation unit passing is at least 75% if true Ag prevalence is 0.5%, and no more than 5% if the true Ag prevalence is ≥1% [2]. In regions with endemic *W*. *bancrofti* and where transmission is dominated by *Aedes* spp. mosquitoes, the TAS threshold is based on an Ag prevalence of 1%. Recent studies have highlighted the limitations of relying solely on Ag-based TAS of young children as a post-MDA surveillance tool, especially as prevalence reaches low levels, and detection of any residual transmission becomes increasingly challenging. For example, in Sri Lanka, TAS of children aged 6–7 years were less sensitive at detecting low-level transmission compared to community-based surveys of people aged ≥10 years, antibody detection in school children aged 6–7 years, or xenomonitoring [6].

In American Samoa, where LF is endemic, *W*. *bancrofti* is the only known species of filarial worm, transmitted by both day and night biting *Aedes* spp. mosquitos. *Ae*. *polynesiensis*, the dominant vector, is highly efficient and day-biting [7]. In 1999, the Ag prevalence using rapid immunochromatographic test (ICT) was estimated to be 16.5% [8, 9]. Under PacELF, the American Samoa Department of Health delivered seven rounds of MDA during 2000–2006. In 2007, Ag prevalence by ICT in a community-based survey had declined to 2.3% with microfilaria prevalence of 0.5% [8, 9]. Another round of MDA was recommended by the WHO Western Pacific Region Technical Advisory group Meeting held in 2008 [10], but was not conducted at large-scale due to logistical reasons [8, 11].

School-based TAS are recommended in regions (e.g. American Samoa) where net school enrolment is ≥75% [2]. The sample size and threshold for TAS are designed to estimate Ag prevalence for the entire evaluation unit. Thus, TAS may not be able to detect small and highly focal residual clusters of transmission, particularly if there is significant spatial variation in prevalence within an evaluation unit. In addition, the age group (6–7 years) tested in TAS is likely to have lower prevalence than older ages, making it even more difficult to detect residual foci.

In American Samoa, TAS-1 (conducted in 2011–2012) identified two Ag-positive children, and TAS-2 (conducted in 2015) identified one Ag-positive child. The Ag-positive children identified during both TAS all attended the same school. As the number of Ag-positive children identified was below the critical cut-off of six, American Samoa passed both TAS-1 [12] and TAS-2 [13].

Despite passing two TAS, recent community-based seroprevalence studies and molecular xenomonitoring studies of mosquitoes provided evidence of low-level but widespread ongoing transmission [14, 15]. In a retrospective study of serum samples collected from adults in 2010, Ag (Og4C3) positive samples were identified from participants living across the main island of Tutuila, with higher Ag prevalence in two localised areas. One of these areas included the school where the Ag-positive children were identified during TAS-1 and TAS-2 [14]. A subsequent study in 2014 confirmed ongoing transmission within the two localised areas by identifying high Ag prevalence and microfilaraemic individuals. In addition, Ag prevalence (ICT) of 1.1% (95% CI 0.2–3.1) was found in children aged 7–13 years who attended the school where Ag-positive children were identified in TAS-1 and TAS-2 [14, 16].

The above findings raise concerns about the suitability of school-based TAS for post-MDA surveillance, not only in American Samoa but globally. In 2016, we conducted a study to compare the effectiveness of two survey designs for post-MDA surveillance: a school-based TAS of children aged 6–7 years and a community-based survey of individuals aged ≥8 years. We also evaluated the use of school-based TAS results as indicators of community-level Ag prevalence and/or foci of ongoing transmission. American Samoa was an optimal study site for answering these operational research questions for two reasons: (i) there had been no MDA since 2007 and any infection in children aged ≤9 years would have been acquired after the last round of MDA, and (ii) evidence from recent studies were highly suggestive of ongoing LF transmission [14–16]. In this paper, we report our key findings and discuss their implications for strengthening of TAS for post-MDA surveillance.

## Methods

### Study location

American Samoa is a US Territory in the South Pacific (14.2710° South, 170.1322° West), consisting of small inhabited islands with a total population of ~55,519 persons living in ~70 villages [17]. Over 90% of the population resides on the main island of Tutuila and the adjacent island of Aunu'u. The remote Manu'a islands were not included in this study as recent seroprevalence studies did not provide any evidence of local LF transmission [14].

### Target population and survey design

This study consisted of two components: A) a school-based survey and B) a community-based survey. Each of the survey designs and sampling methods are described below.

#### School-based survey

Based on WHO TAS guidelines [2], a systematic school-based survey was used to sample children aged 6–7 years from all elementary schools (N = 29) on the two main islands of Tutuila and Aunu'u (hereafter referred to as American Samoa), where >95% of the population reside [2, 12]. Attendance at Grade 1 and 2 in elementary school was used as proxy for being 6–7 years old. Assuming 1% Ag prevalence, the target sample size calculated using the *Survey Sample Builder* [18] was 1,014 children and the critical cut-off value for failing TAS was six or more Ag-positive children.

#### Community-based survey

In parallel with the school-based survey, a multi-stage equal probability cluster survey based on WHO guidelines was conducted [2]. For the village selection process, large villages were divided into village segments of <2000 residents and very small adjacent villages were grouped. The 70 villages/village segments/village groups (primary sampling units, PSUs) were ranked in order of population size, and 30 were selected based on a randomly selected starting point and sampling interval of 2.33.

Using *Survey Sample Builder*, the sample size required to detect Ag prevalence of 1% was estimated to be 4,620 for persons aged ≥8 years. We assumed a target population of ~55,000 persons and accounted for an additional 1.5 times within household clustering of participants.

The total numbers of households in the selected villages were estimated based on the most recent census data and an average of seven persons per household. The target number of households was estimated by dividing the target sample size of persons by the average household size of seven and accounting for a 15% non-response/ absentee rate. A sampling fraction was calculated as the proportion of households that needed to be sampled to achieve the target sample size. In each PSU, 29% households were selected (sampling fraction of 0.29).

Within each PSU, households were randomly selected from a line list of geo-referenced buildings obtained from the American Samoa Department of Commerce [19]. Detailed village maps showing locations and codes of selected households were prepared and printed prior to fieldwork, and used during village visits to identify the selected houses. Destroyed, abandoned or currently unoccupied households were substituted with the next closest inhabited household. Within each household, all members aged ≥8 years were invited to participate. A household member was defined as an individual who considered the selected house as their principal place of residence or who slept in that house the previous night.

For both school and community-based surveys, participants were eligible irrespective of previous participation in MDA, duration of school attendance, or length of residence in their current villages or in American Samoa.

### Informed consent, ethics approvals, and cultural considerations

For the school-based survey, information sheets and consent forms were distributed to parents/guardians of all Grade 1 and 2 children approximately one week prior to scheduled school visits. All children with valid consent forms were included, and assent was sought from all participants. For the community-based survey, signed informed consent was obtained from adult participants or from parents/ guardians of those aged <18 years, along with verbal assent from minors.

Ethics approvals for the study were granted by American Samoa Institutional Review Board and the Human Research Ethics Committee at the Australian National University (protocol number 2016/482). The study was conducted in collaboration with the American Samoa Department of Health and the American Samoa Community College. Official permissions for school and village visits were granted by the Department of Education and the Department of Samoa Affairs, respectively. All field activities were carried out in a culturally appropriate and sensitive manner with bilingual local field teams, and with verbal approval sought from village chiefs/ mayors prior to conducting the community surveys. Surveys were conducted in English or Samoan depending on the participants’ preference. The Institutional Review Board of the U.S. Centers for Disease Control and Prevention (CDC) determined CDC to be a non-engaged research partner.

### Data and sample collection

#### School-based survey

At each school, we recorded the geographic positioning system (GPS) coordinates for the location of the school, total number of children enrolled in Grades 1 and 2, and the school attendance for the day. Along with the consent forms, parents/guardians were asked to complete a short questionnaire about the child, including age, sex, place of birth, village of residence, and the number of years lived in the current village. These demographic data were coded into electronic questionnaires during the school visits.

#### Community-based survey

**Fig 1:**
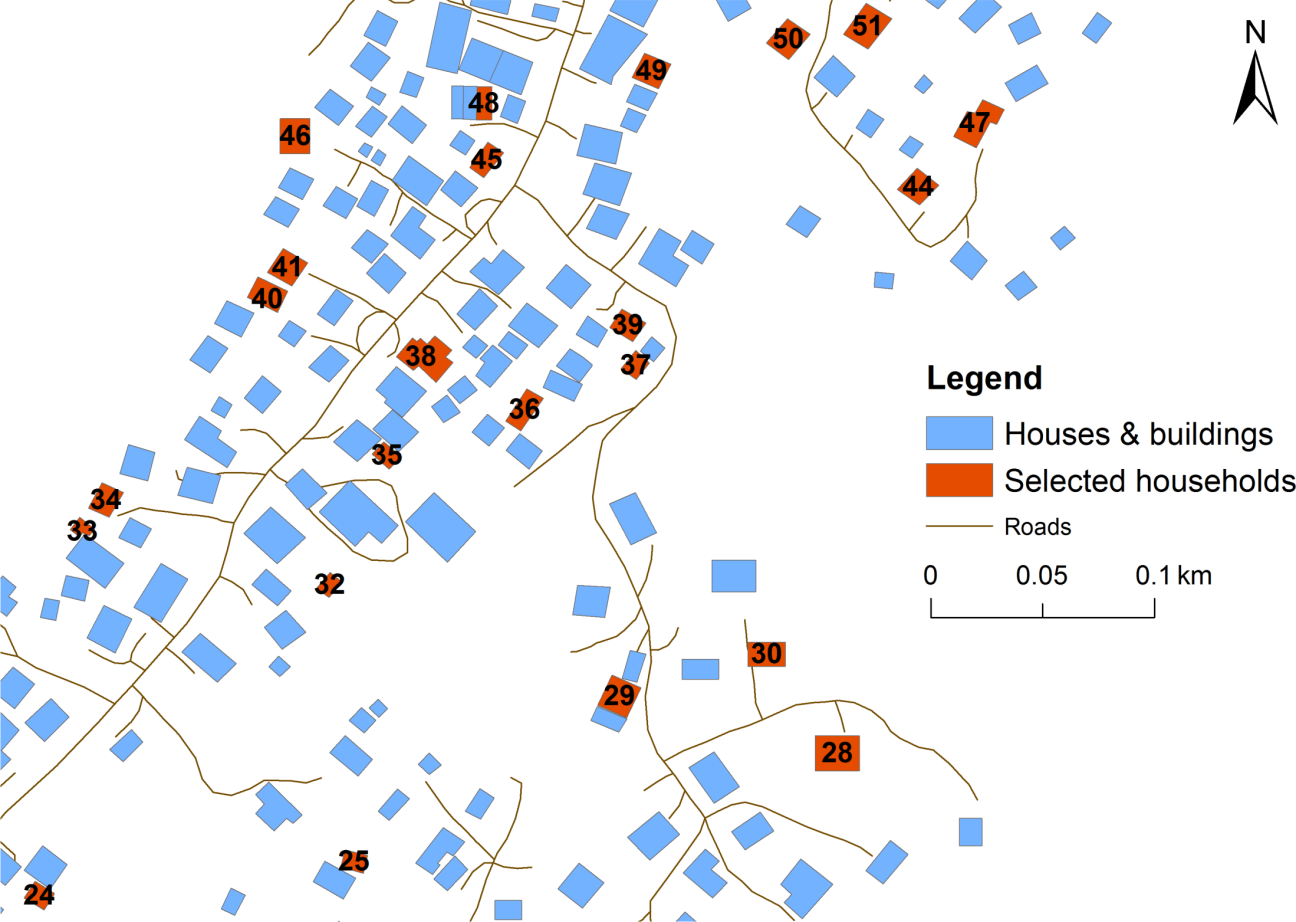
Sample village map used to identify selected households during fieldwork, American Samoa, 2016.

Selected households were identified using fieldwork maps as described above (Fig 1). GPS locations of households were recorded at the time of visit. If GPS satellite signal was not available or a household was substituted, the location was marked on a map, and reconciled manually using the geographic information systems software (ArcGIS v10.4.1, Environmental Systems Research Institute, Redlands CA). On every occasion that we visited a village, we attempted to revisit households with previously-absent members to maximise participation rates.

Electronic questionnaires were administered by bilingual field research assistants in Samoan or English according to each participant’s preference. Questions included demographics, occupation, number of household members aged ≥8 years, country of birth, duration lived in American Samoa, travel history and history of taking medications during MDA. Data were recorded using smart phones, utilising the LINKS electronic database system developed by the Task Force for Global Health [20].

#### Specimen collection and testing

For each participant, we collected 200μl of finger prick blood sample into heparinised microtainers. The blood samples were kept cool and tested on the same or following day in a controlled laboratory environment at the American Samoa Community College or the Department of Health Public Health Laboratory. Blood samples were tested for circulating filarial antigen (CFA) using the Alere Filariasis Test Strip (FTS) [21–23]. Results were recorded as positive, negative, or invalid and linked to each individual’s survey data using the LINKS system.

#### Follow up of Ag-positive individuals

All Ag-positive school children were followed-up at home and treated with weight-appropriate doses of albendazole and diethylcarbamazine (DEC) in the presence of a parent or guardian. Household members of Ag-positive children were invited to participate in the survey (using the same protocol as the community-based survey) unless the child’s household was already selected for the community-based survey. All Ag-positive community participants (excluding pregnant women) were invited to a follow-up clinic, where they were given treatment with 400mg albendazole and 6mg/kg DEC according to WHO guidelines [2]. To ensure compliance, participants were encouraged to consume their medications in the presence of a field team member. All minors aged <18 years were given treatment following parental/ guardian consent.

#### Microfilaria slides

**Fig 2:**
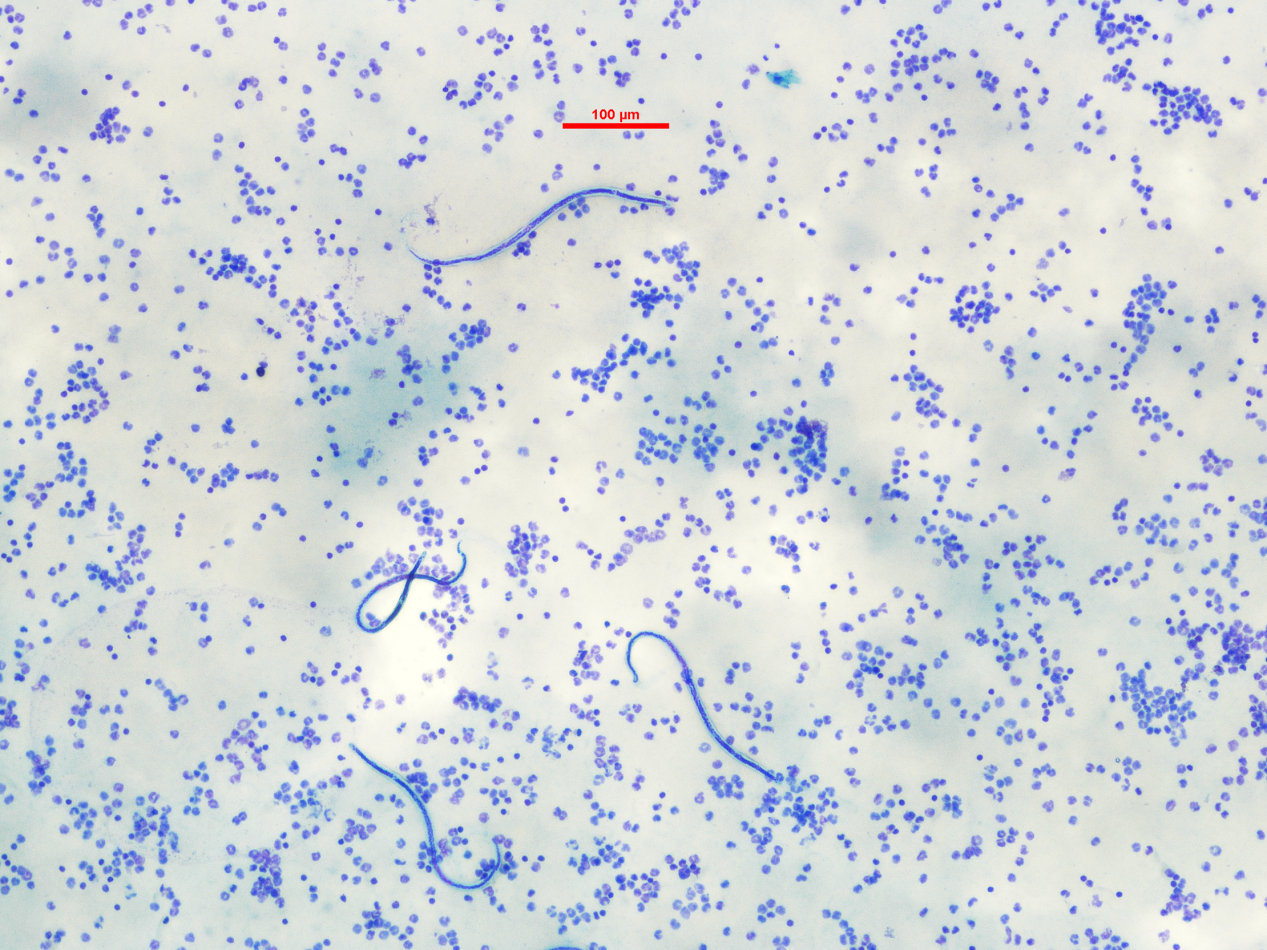
Blood film with microfilaria from infected study participant, American Samoa, 2016.

During follow-up of Ag-positive people, we collected venous blood samples with heparin anticoagulant (~8ml) to repeat the FTS and prepare slides for microscopic examination of microfilaria (Mf) as described previously [16]. As filarial worms in the South Pacific region are diurnal sub-periodic [24, 25], all blood samples were collected during the day. Briefly, we prepared three slides per person, by applying three lines of 20μl of blood to each slide. Once completely dried, the slides were de-haemoglobinized, fixed with methanol and stained with 2% Giemsa stain for 50 minutes. Each set of three slides was examined by two or three experienced parasitologists in American Samoa and Australia. A slide was considered Mf positive if ≥1 microfilaria were identified by at least one parasitologist (Fig 2). The average of counts reported by all parasitologists was used to calculate the final density in Mf/ml.

#### Data analyses

The outcome measure was a positive FTS test. We undertook descriptive analyses for questionnaire variables and compared simple proportions using Pearson’s chi square tests or Fisher exact tests. We estimated crude CFA prevalence and 95% confidence intervals (CI) using binomial exact methods.

Prevalence estimates were adjusted for survey design, and age and sex distribution. We accounted for the multi-stage cluster sampling design of the survey using the ‘svyset’ command in Stata 13 (StataCorp, 24 College Station, TX). Design effect was calculated using the ‘deff’ command. For the school-based survey, we calculated sampling weight for each participant by adjusting for participation rates by schools, and applied post-stratification weights for sex to the entire sample. For the community-based survey, we calculated a sampling weight for an individual as the inverse product of the probability of selection of the PSU, household and individual. We weighted for absentees within households and for coverage within each village to account for those households which could not be surveyed either due to logistical reasons, non-response or were vacant at the time of village visits. As 30 out of the 70 eligible PSUs were selected, and selection was done without replacement, we applied a finite population correction (FPC) factor of 30/70 [26, 27]. To estimate the country- and village-level CFA prevalence for people aged ≥8 years, we applied post-stratification weights for age and sex based on American Samoa’s demographic distribution using information available from the 2014 Statistical Yearbook [17].

We evaluated the utility of school-based TAS results for identifying villages with different levels of seroprevalence, using thresholds of 1%, 2%, 5%, 10% and 20%. We calculated the sensitivity, specificity, positive predictive value (PPV) and negative predictive value (NPV) of each of the following findings from the school-based TAS for identifying villages with seroprevalence above those thresholds: i) villages of residence of all Ag-positive children; ii) villages of school location all Ag-positive children; and iii) villages of residence AND villages of school location of all Ag-positive children. In other words, if we did not conduct a community-based survey, and used the results of the school-based TAS as potential indicators of high prevalence villages, how well would we have performed?

Population estimates for American Samoa were sourced from the American Samoa Statistical Yearbook and were based on the 2010 census [17]. All analyses were performed using Stata 13 or Microsoft Excel, and *p* values of <0.05 were considered statistically significant.

## Results

We recruited a total of 3650 participants, including 1143 and 2507 persons from the school-based and community-based surveys respectively (Table 1).

**Table 1:** Summary of TAS strengthening in American Samoa, 2016.

Survey demographics	Number recruited	Number of valid FTS^1^ (%)	Number FTS positive	Crude CFA prevalence (%)	Adjusted CFA prevalence (95% CI)	Number of individuals with microfilariae slides collected^2^	Number of individuals with microfilariae positive slides (%)
**A. School-based survey**	1143	1143 (100)	9	0.8	0.7 (0.3–1.8)^3^	9	1 (11.1)
**B. Community-based survey**	2507	2496 (99.6)	102	4.1	6.2 (4.5–8.6) ^4^	86	22 (25.6)
**All participants**	3650	3639 (99.7)	111	-	-	95	23 (24.2)

^1^ FTS results were classified as invalid if the test result was invalid or if there was insufficient blood sample to conduct the test

^2^ Excludes Ag-positive individuals who were lost to follow-up or did not want to be bled at time of follow-up

^3^ Adjusted for survey design and sex using ‘svyset’ in Stata13.

^4^ Adjusted for survey design, age and sex using ‘svyset’ in Stata13

### School-based TAS of Grade 1 and 2 children

We included all elementary schools (N = 29) on the two main islands of Tutuila and Aunu’u. Of 2180 Grade 1 and 2 students who were eligible to participate, 1143 (52.4%) students returned signed consent forms and were included in the study. The average participation rate by school was 57% (range 18.2–91.7%).

Table 2 summarises characteristics of participants included in the school-based TAS. Of the 1143 students, we identified nine Ag-positive children, equivalent to a crude CFA prevalence of 0.8%. As the critical cut-off for passing TAS was fewer than six Ag-positive children, American Samoa failed the school-based TAS.

**Table 2:** Summary of participants in the school-based survey, American Samoa, 2016.

Questionnaire variables	Number tested (% of total tested)	Number Ag- positive (Crude CFA prevalence)	*p* value^1^
**Total**	1143 (100)	9 (0.8)	
**Age (years)**			
5	62 (5.4)	0 (0)	0.74
6	524 (45.8)	3 (0.6)	
7	510 (44.6)	5 (1.0)	
8	39 (3.4)	1 (2.6)	
9	6 (0.5)	0	
10	2 (0.2)	0	
**Sex**			
Male	550 (48.1)	3 (0.5)	0.373
Female	593 (51.2)	6 (1.0)	
**Location of school**			
Nua	44 (3.9)	2 (4.5)	**<0.001**
Pago Pago	82 (7.2)	4 (4.9)	
Ili'ili	94 (8.2)	1 (1.1)	
Nu'uuli	93 (8.1)	1 (1.1)	
Faga'alu	44 (3.9)	1 (2.3)	
Others	786 (68.8)	0 (0)	
**Place of birth**			
American Samoa	1000 (87.5)	7 (0.7)	**0.037**^**2**^
Samoa	54 (4.7)	2 (3.7)	
Other^3^	83 (7.3)	0 (0)	
Unknown^4^	6 (0.5)	0 (0)	
**Village of residence**			
Faga'alu	16 (1.4)	1 (6.3)	**<0.001**
Fagali'i	7 (0.6)	2 (28.6)	** **
Fagatogo	37 (3.2)	2 (5.4)	
Pago Pago	73 (6.4)	2 (2.7)	
Tafuna	157 (13.7)	1 (0.6)	
Vaitogi	55 (4.8)	1 (1.8)	
All other villages	798 (69.8)	0 (0)	
**Duration lived in the village**			
Less than 1 year	61 (5.3)	1 (1.6)	0.723
1–2 years	79 (6.9)	1 (1.3)	
3–5 years	154 (13.5)	0 (0)	
≥6 years	845 (73.9)	7 (0.8)	
Unknown	4 (0.4)	0 (0)	

^1^ *P* value estimated using Chi-square or Fisher exact for significance of difference in crude CFA prevalence between subgroups. Statistically significant results are highlighted in bold.

^2^ *P*-value comparison excludes unknown place of birth.

^3^ Other countries of birth include the United States of America (mainland), Hawaii, New Zealand and other Pacific Island Countries.

^4^ Data not provided by parent at time of consent.

The estimated overall CFA prevalence after adjusting for participation rates by school and sex was 0.7% (95% CI: 0.3–1.8). The design effect for the school-based survey was 1.9. Adjusted CFA prevalence in males was 0.5% (95% CI: 0.1–1.9) and in females was 0.9% (95% CI: 0.4–2.4).

Valid FTS results were available for all 1143 (100%) children. Of the nine Ag-positive children, four (44.4%) attended the same school in Pago Pago, and two (22.2%) attended the same school in Nua. The other three Ag-positive children attended different schools located in the villages of Ili'ili, Nu'uuli and Faga'alu. Both Ag-positive children from the school in Nua lived in Fagali’i, one of the suspected hotspots identified in previous studies [14, 16]. Seven (77.8%) Ag-positive children were born in American Samoa and reported to have lived there for their entire lives, and two (22.2%) were born in Samoa.

Of the nine FTS-positive children, one (11.1%) was microfilaraemic with Mf density of 1075 Mf/ml. The child was a 7 year old male who lived in Vaitogi, another potential hotspot identified in previous studies and attended the school where Ag-positive children were identified in TAS-1 and TAS-2.

### Community-based survey of selected villages

We visited all 30 selected PSUs and sampled 2507 persons from 711 households. The villages of Ili’ili and Pava’ia’i were split into two segments during the selection process, and both segments of these villages were selected for the survey. For the purposes of analyses, data from the different segments of each village were pooled, and results were presented for 28 villages (comprising 30 PSUs).

The average household size was 6 (range 1–25) persons aged ≥8 years. We recruited participants from 77.6% of the selected households, and 83.2% (range 14.3–100%) of eligible household members (aged ≥8 years). On average, 16.8% of eligible household members were not recruited, and non-response was mostly associated with household members being absent at the time of visit, rather than refusal to participate. We recruited 1,140 (45.5%) males and 1,367 (54.5%) females (Table 3). Of the 2507 participants tested, 11 (0.4%) had invalid tests and were excluded from analyses (Tables 1 & 3). Of the 2496 participants with valid tests, 102 were Ag-positive, equivalent to an overall crude CFA prevalence of 4.1%. Of the 102 FTS-positive persons, 79 were male (crude CFA prevalence 7.0%) and 23 were female (crude CFA prevalence 1.7%), and this difference was statistically significant (p<0.001).

**Table 3:** Summary of participants in the community survey, American Samoa, 2016.

Questionnaire variables	Number tested (%)	Number Ag-positive (Crude CFA prevalence %)	*p* value^1^
**Total**	2496 (100.0)	102 (4.1)	
**Age group (years)**			
8 to 9	147 (5.9)	4 (2.7)	**<0.001**
10 to 19	732 (29.2)	6 (0.8)	
20 to 29	363 (14.5)	8 (2.2)	
30 to 39	315 (12.6)	18 (5.7)	
40 to 49	340 (13.6)	22 (6.5)	
50 to 59	309 (12.4)	25 (8.1)	
60 to 69	183 (7.3)	9 (4.9)	
≥70	107 (4.3)	10 (9.3)	
**Sex**			
Male	1130 (45.3)	79 (7.0)	**<0.001**
Female	1366 (54.7)	23 (1.7)	

^1^ *P* value estimated using Chi-square or Fisher exact for significance of difference in crude CFA prevalence. Statistically significant results are highlighted in bold.

The original target sample size for the community-based survey, calculated based on an expected CFA prevalence of 1%, was 4620. After the first two weeks of recruitment, the observed CFA prevalence (~4%) was significantly higher than anticipated, and it was determined that a smaller target sample size of 2981 would provide adequate statistical power (Table 4).

**Table 4:** Summary of sampling and recruitment for community survey; and prevalence of circulating filarial antigen (CFA) for selected villages, American Samoa 2016.

Village	Total number of residents^1^	Total number of households	Target number of households	Number of households sampled (% of target)	Target population aged ≥8 years	Number recruited (% of target)	Number FTS-positive	Number Microfilaria positive	Crude CFA prevalence (%)	Adjusted CFA prevalence (95% CI)^2^
Afono	524	75	22	21 (95.5)	69	71 (82.3)	3	1	4.2	4.0 (1.7–9.3)
Alao	495	71	20	12 (60)	65	44 (54)	0	-	0	-
Amaua	96	14	4	5 (125)	13	19 (120.2)	1	-	5.3	4.9 (1.4–16.3)
Amouli	920	131	38	33 (86.8)	121	111 (73.3)	2	-	1.8	2.7 (1–7)
Asili	224	32	9	9 (100)	30	28 (75.9)	4	-	14.3	19.6 (9.7–35.6)
Auma	254	36	10	9 (90)	33	39 (93.2)	2	-	5.1	8.3 (3.2–19.7)
Aumi	186	27	8	6 (75)	25	23 (75.1)	0	-	0	-
Fagamalo	47	7	2	3 (150)	6	13 (168)	4	3	30.8	47.1 (16.9–79.6)
Faganeanea	150	21	6	5 (83.3)	20	23 (93.1)	0	-	0	-
Fagatogo	1737	248	72	55 (76.4)	229	212 (74.1)	5	-	2.4	2.7 (1.4–5.2)
Fatumafuti	113	16	5	3 (60)	15	5 (26.9)	1	-	20.0	44.8 (10–85.5)
Ili'ili^3^	3195	456	132	87 (65.9)	421	308 (58.5)	15	3	4.9	4.9 (3.2–7.5)
Lauli'i	892	127	37	27 (73)	118	104 (70.8)	1	-	1.0	1.1 (0.3–4)
Leloaloa	448	64	18	15 (83.3)	59	40 (54.2)	7	2	17.9	25.8 (16.1–38.4)
Malaeimi	1182	169	49	36 (73.5)	156	120 (61.6)	5	1	4.2	10.9 (5–22.2)
Malaeloa/Aitulagi	698	100	29	20 (69)	92	90 (78.3)	4	2	4.4	8.1 (3.3–18.6)
Masausi	164	23	7	7 (100)	22	24 (88.9)	0	-	0	-
Nua	141	20	6	3 (50)	19	17 (73.2)	0	-	0	-
Pago Pago^4^	1828	261	75	62 (82.7)	241	228 (75.7)	4	1	1.8	2.3 (1.2–4.5)
Pava'ia'i	2450	350	101	73 (72.3)	323	255 (63.2)	3	-	1.2	2.5 (0.8–7.5)
Satala-Anua-Atuu	674	96	28	22 (78.6)	89	81 (73)	7	2	8.8	9.0 (4.3–17.7)
Se'etaga	299	43	12	13 (108.3)	39	49 (99.5)	2	-	4.1	3.4 (1.3–8.5)
Tafuna^5^	2000	286	82	56 (68.3)	263	187 (56.8)	5	1	2.7	3.3 (1.4–7.6)
Taputimu	841	120	35	29 (82.9)	111	88 (63.5)	0	-	0.0	-
Tula	405	58	17	14 (82.4)	53	52 (78)	4	-	7.7	14.5 (5.9–31.5)
Utumea West	53	8	2	3 (150)	7	12 (137.5)	1	-	8.3	12.7 (3.2–39.4)
Vaitogi	1959	280	81	64 (79)	258	212 (65.7)	18	6	8.5	11.8 (7.9–17.4)
Vatia	640	91	26	19 (73.1)	84	52 (49.3)	4	-	7.8	21.8 (9.8–41.6)
**Total**	**22601**	**3230**	**933**	**711 (76.2)**	**2981**	**2507 (84.1)**	**102**	**22**	**4.1**	**6.2 (4.5–8.6)**

^1^ Population estimates based on American Samoa 2014 Statistical Yearbook (American Samoa Department of Commerce). 80% of the population is estimated to be aged ≥8 years.

^2^ Adjusted for survey design, and post-stratified for age and sex using ‘svyset’ in Stata13.

^3^ Villages were split into two segments for the selection of PSUs. Both segments of both villages were selected. Data presented here are pooled for both segments for each of the villages.

^4^ One of two segments of Pago Pago was selected; number of residents shown here is half of the total population of Pago Pago.

^5^ One of four segments of Tafuna was selected; number of residents shown here is quarter of the total population of Tafuna.

**Fig 3:**
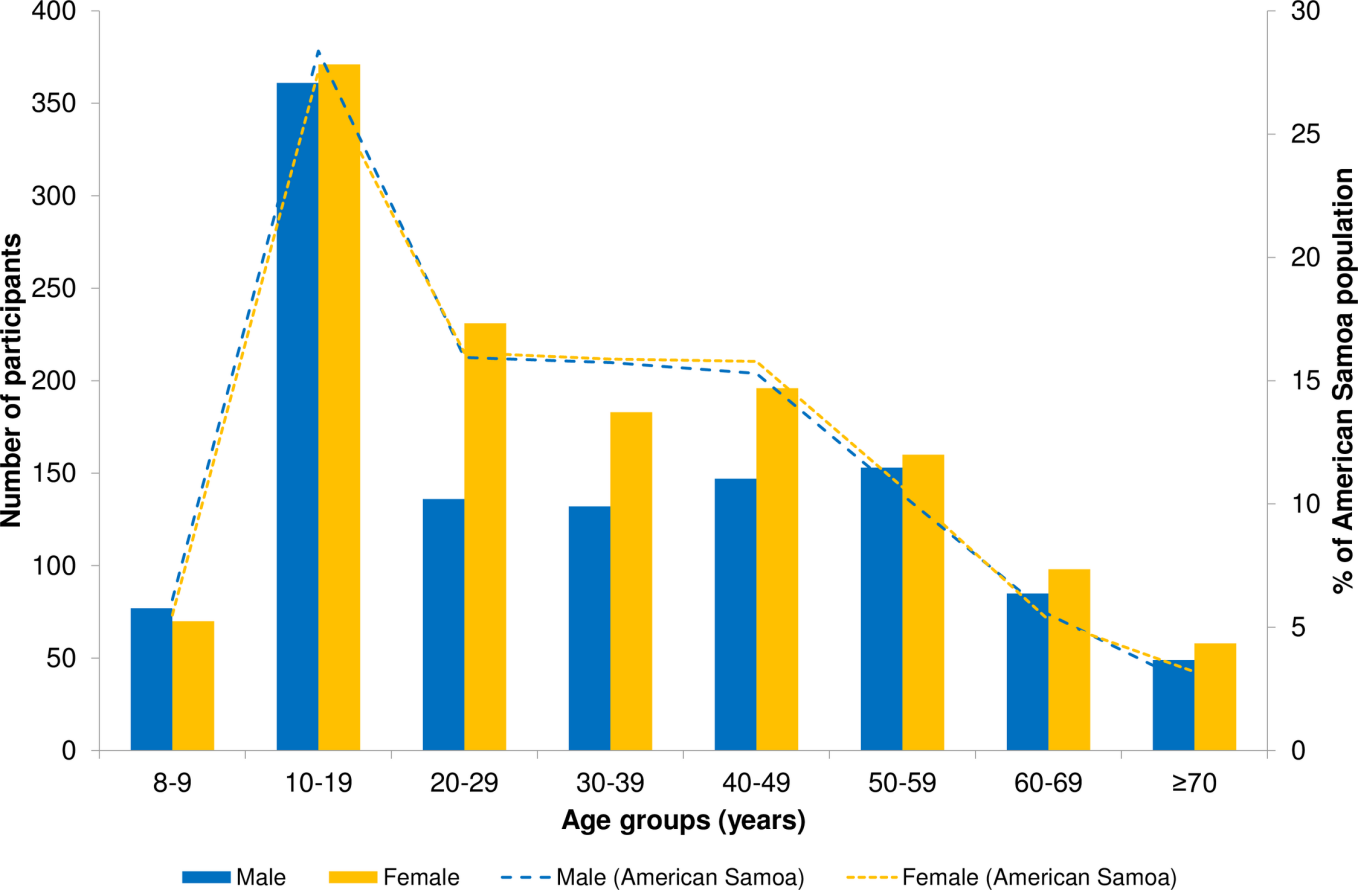
Age and sex distribution of participants (bars) from community survey and general population (dotted lines) living in American Samoa, 2016. Population estimates based on American Samoa 2014 Statistical Yearbook (American Samoa Department of Commerce).

**Fig 4:**
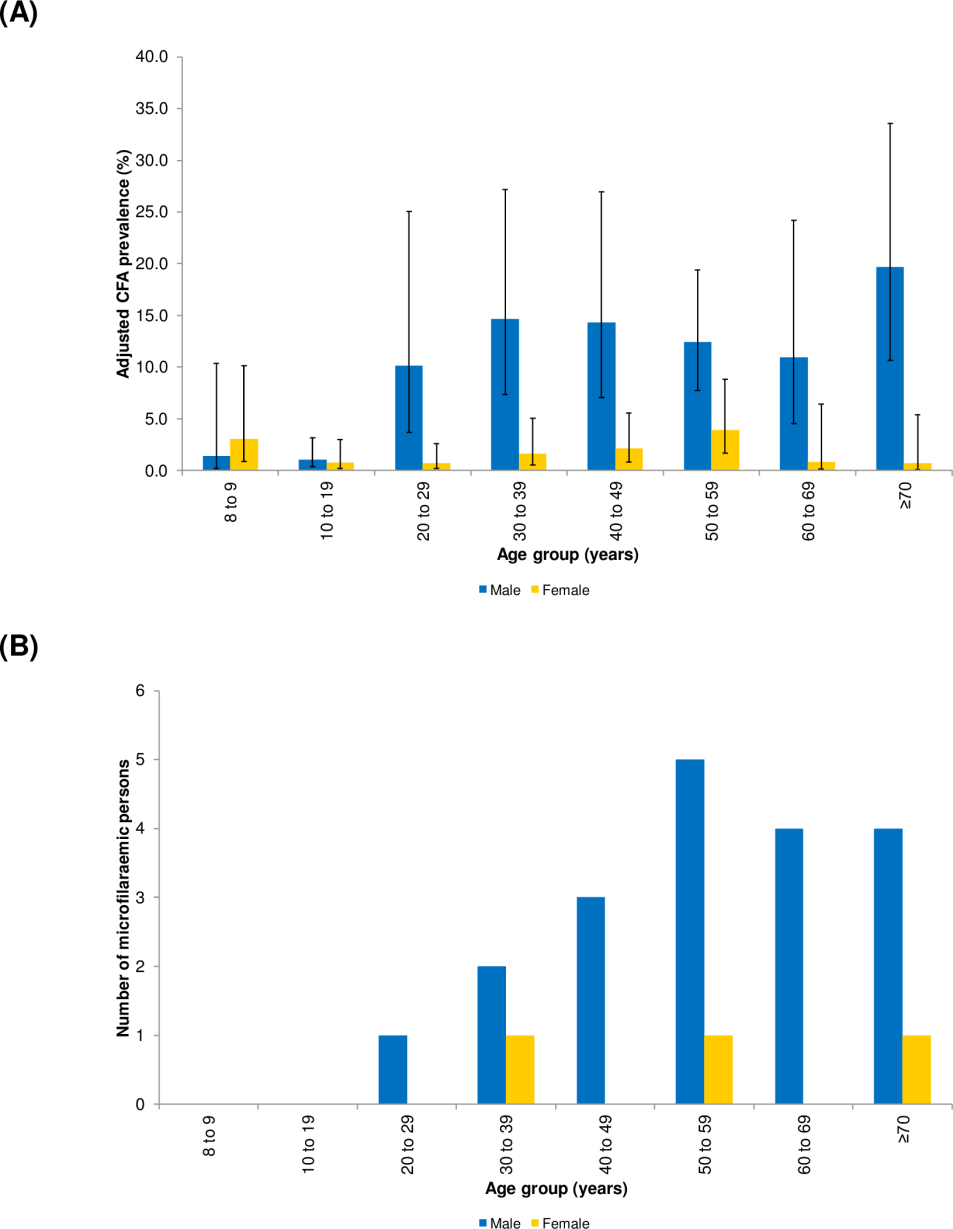
Adjusted* circulating filarial antigen (CFA) prevalence with 95% CIs [A] and microfilaraemic individuals by age and sex in community survey [B], American Samoa 2016. *Adjusted for survey design.

The age and sex distribution of the community-based survey participants and the general population of American Samoa are presented in Fig 3, showing that the survey included proportionately more females but provided a good representation of all age groups. After adjusting for the survey design, and age and sex distribution of American Samoa, the overall adjusted CFA prevalence was 6.2% (95% CI: 4.5–8.6). The design effect for the community-based survey was 4.2. The adjusted CFA prevalence by age and sex in the selected villages are presented in Fig 4A. Notably, in children aged 8–9 years, who were born after MDA had stopped, the adjusted CFA prevalence was 2.2% (95% CI: 0.8–6.1).

Of the 102 FTS-positive individuals, we were able to prepare slides for 86 (84.3%) participants. Of these, 22 (25.6%) were microfilaraemic, of whom 19 (86.4%) were male (Fig 4B). The geometric mean Mf density was 60.7 Mf/ml (range 5.6–916.7 Mf/ml).

**Fig 5:**
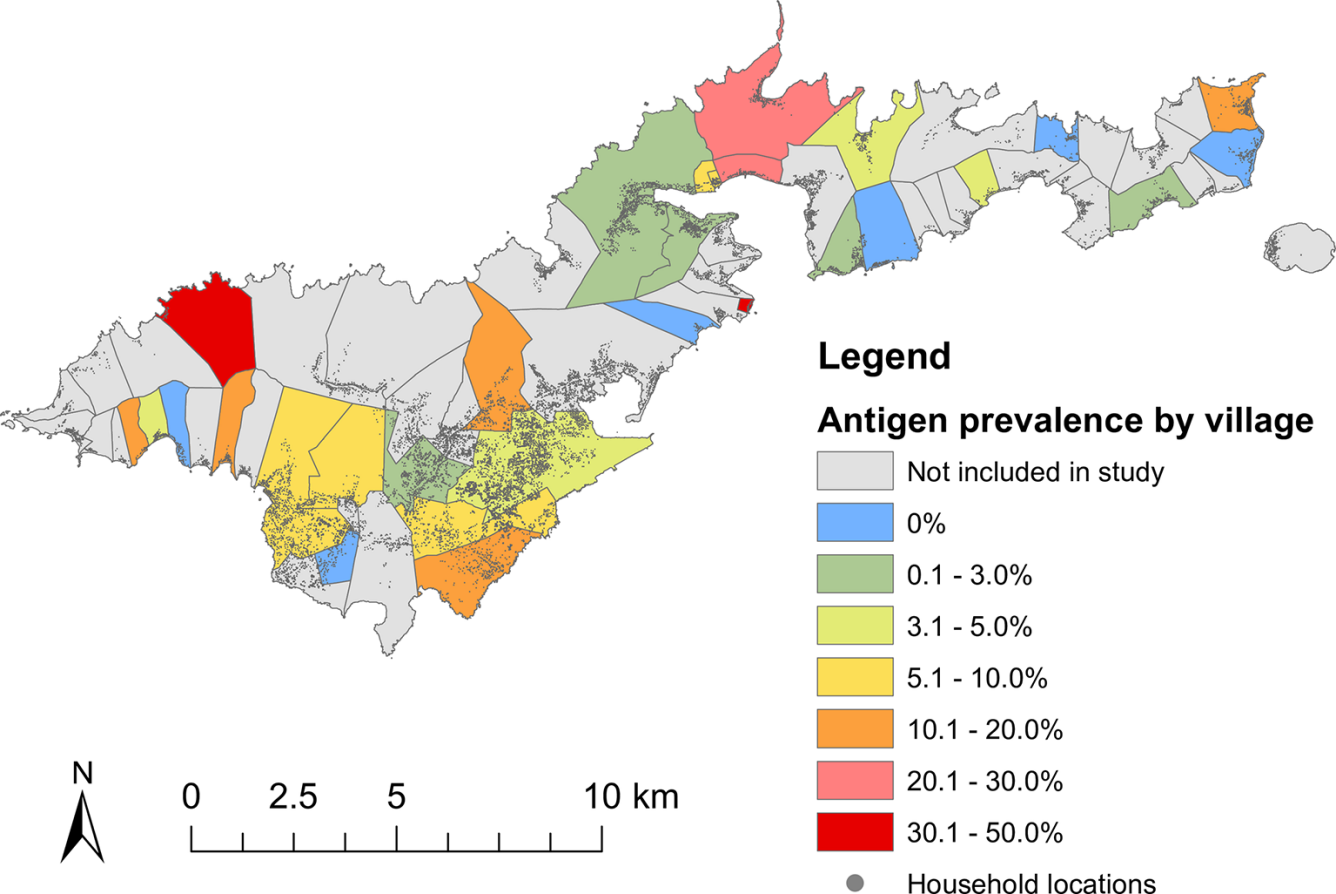
Location of selected villages (N = 28) and adjusted* circulating filarial antigen (CFA) prevalence, American Samoa, 2016. *Adjusted for survey design, age and sex distribution of American Samoa. To illustrate the distribution of the general population across American Samoa, locations of households (grey dots) are also shown.

Adjusted village-level CFA prevalence varied from 0% to 47.1% (Table 4 and Fig 5). Of the 28 villages, Ag-positive individuals were identified in the majority of villages, and only 6 (21.5%) villages had no Ag-positive individuals. Microfilaraemic people identified from the community survey were dispersed throughout the island and lived in 10 of the 28 selected villages. Of the 22 microfilaraemic people, six (27.3%) lived in Vaitogi (focal area with ongoing transmission identified in previous studies), the same village as the Mf-positive school child.

### Utility of school-based TAS results for identifying villages with high seroprevalence

Ag-positive school children from the school-based survey lived in four (14.3%) of the 28 villages included in the community-based survey and attended school in three villages (10.7%). A total of six villages (21.4%) were identified as communities where Ag-positive children lived and/or attended school. Three (33.3%) and two (22.2%) Ag-positive children identified in the school-based survey, respectively, lived in and attended school in villages that were not selected for this survey; these data were therefore not included in the analysis of sensitivity, specificity, PPV and NPV. Table 5 shows the sensitivity, specificity, PPV and NPV for using Ag-positive children as indicators of villages with adjusted CFA prevalence of greater than 1%, 2%, 5%, 10%, and 20%. These findings suggest that follow-up of Ag-positive school children would have identified areas of transmission with high specificity (>73% for all scenarios), but low sensitivity (<24% for all scenarios), even if we considered villages where Ag-positive lived and/or attended school. Ag-positive children were a poor indicator of villages with higher prevalence (10 or 20%), with PPV of 25% or less for any of the scenarios tested.

**Table 5:** Sensitivity, specificity, positive predictive value (PPV), and negative predictive value (NPV) of the presence of antigen (Ag) positive children from school-based survey as indicators of villages with 1%-20% adjusted* CFA-prevalence.

Indicator	Adjusted CFA-prevalence in villages	Sensitivity (%)	Specificity (%)	PPV (%)	NPV (%)
**A. Villages where** **Ag-positive school children lived**^**1**^ **(N = 4)**	1%	18.2	100.0	100.0	25.0
	2%	19.0	100.0	100.0	29.2
	5%	7.7	80.0	25.0	50.0
	10%	11.1	84.2	25.0	66.7
	20%	0	83.3	0	83.3
**B. Villages where** **Ag-positive school children attended school**^**2**^ **(N = 3)**	1%	9.1	83.3	66.7	20.0
	2%	9.5	85.7	66.7	24.0
	5%	7.7	86.7	33.3	52.0
	10%	0	84.2	0	64.0
	20%	0	87.5	0	84.0
**C. Villages where** **Ag-positive children lived and/or attended school** **(N = 6)**	1%	22.7	83.3	83.3	22.7
	2%	23.8	85.7	83.3	27.3
	5%	15.4	73.3	33.3	50.0
	10%	11.1	73.7	16.7	63.6
	20%	0.0	75.0	0	81.8

*Adjusted for survey design, and age and sex distribution.

^1^ Three antigen-positive children lived in two villages which were not selected for the community survey, and were excluded from analyses.

^2^ Two antigen-positive children attended school in two villages which were not selected for the community survey and were excluded from analyses.

Grey: 0–25.0%, light blue: 25.1–50.0%, medium blue: 50.1–75.0%, dark blue: 75.1–100%.

## Discussion

Our study confirmed recrudescence of LF transmission in American Samoa 10 years after the last round of MDA. Through the school survey, we identified nine Ag-positive children (adjusted CFA prevalence 0.7%), including a seven-year-old microfilaraemic child. The community survey identified 102 Ag-positive persons (adjusted CFA prevalence 6.2%) and 22 microfilaraemic individuals. American Samoa failed school-based TAS in 2016, and the community-based survey identified higher numbers of Ag-positive people compared to Ag prevalence of 2.3% (by ICT) in 2007 and 3.2% (by Og4C3 Ag) in 2010 [8, 14]. The adjusted CFA prevalence in the school-based survey of children aged 6–7 years (0.7%) was significantly lower than the community-based survey of people aged ≥8 years (6.2%), consistent with our knowledge that CFA prevalence is generally higher in older age groups.

Our study identified advantages and limitations of using school-based versus community-based surveys. The school-based survey was logistically simpler, faster and cheaper, while the community-based survey was practically more challenging and time-consuming but provided more detailed information on estimates of community-level CFA prevalence, highlighted foci of high prevalence, and identified Ag- and Mf-positive people who are capable of perpetuating transmission. With the school-based survey, the identification of Ag-positive young children who have lived in American Samoa for their whole lives provided clear evidence of transmission within the past 6–7 years. The community survey also provided evidence of recent transmission by identifying Ag-positive children aged 8–9 years (adjusted CFA prevalence of 2.2%, 95% CI 0.8–6.1), who were either born after the last round of MDA in 2008 or were too young to participate. In addition, the community-based survey provided detailed epidemiological information for a wider age range, including identification of older Ag-positive people who may serve an important reservoir of parasites and maintain transmission in the post-MDA setting [28]. Our results indicate that TAS conducted among young children (who have lower seroprevalence) may not be sufficiently sensitive to identify all areas of residual transmission (i.e where Ag-positive people aged ≥8 years were identified). This finding supports the conclusions of a recent study which modelled the efficiency of surveillance protocols based on the combination of ability to identify transmission foci, sample size required and high PPV, and found that testing of adults would be more efficient at detecting transmission in low prevalence settings compared to testing children aged 6–7 years [28].

The school-based TAS did not identify any difference in CFA prevalence between male and female young children. The community-based survey indicated that males (particularly those aged ≥20 years) had higher CFA prevalence and greater proportion of those who were Ag-positive had detectable microfilaria. Higher prevalence in adult males could be related to more time spent outdoors for work and recreation, and/or lower rates of participation in MDA [14, 29]. Hormonal and pregnancy-mediated regulation of the immune system may also contribute to lower infection rates in females, particularly during the reproductive years [30].

The school-based survey was a systematic survey where all elementary schools on the two main islands were included. Overall, 52.4% of our target population (6–7 year olds) participated in the survey, and we do not have reasons to suspect differences in Ag prevalence amongst those children who did not participate. The school-based survey identified two FTS-positive children living in Fagali’i, an area of known high LF transmission [16], and a cluster of four FTS-positive children who attended one school and lived in Fagatogo and Pago Pago. Considering that both villages of residence had low estimated CFA prevalence of 2.7% and 2.3%, respectively (below the overall estimated CFA prevalence of 6.2%), the school-based clustering raises the possibility that transmission might be occurring in and around the school, particularly in the presence of a highly efficient day-biting vector [7]. The school-based survey had limited utility for detecting focal areas of ongoing transmission, (Table 5); while follow-up of villages where Ag-positive children lived or went to school might help identify areas of transmission with high specificity (range 83.3–100%), the low sensitivity (range 0–23.8%), PPV (0% in villages with >20% Ag prevalence) and NPV (range 25–83.3%) suggests that even if further surveillance was conducted in all the villages where Ag-positive children lived and/or attended school, many high prevalence villages would still have been missed.

The community-based survey was a modified WHO cluster survey, which is recommended for surveying large populations in resource-limited settings. By using a population representative sampling design and correcting for clustering during analyses [27, 31], we believe our results are an accurate estimate of the country-wide CFA prevalence. The community-based survey demonstrated significant heterogeneity in CFA prevalence between villages, even in the very small and isolated island. Similar observations were made in the 2007 survey in American Samoa, and during post-MDA surveillance studies in other small island countries such as Sri Lanka and Samoa [6, 8, 32]. A limitation of the survey design was that the school and community-based surveys were not completely geographically aligned because all schools were included, but only 30 of the 70 PSUs were sampled, i.e. some children tested in the school-based survey lived in villages that were not selected for the community-based survey. However, as we surveyed a large proportion of the selected villages, and many villages are contiguous along the limited number of roads in American Samoa (Fig 5), geographical concordance between the two surveys is unlikely to be a major issue in this study.

The reasons for recrudescence of LF in American Samoa are unclear but could be associated with a combination of factors including some areas of poor-coverage or systematic non-compliance during MDAs [33, 34], travel and migration of people from other countries in the Pacific where LF transmission is still ongoing [14, 29, 34]. An outdoor lifestyle in the presence of highly efficient day and night biting mosquitoes could also have contributed to recrudescence, and lower target thresholds may need to be considered in such settings [7, 15].

LF has a long prepatent period [35], leading to low prevalence in young children even when prevalence is high in adults [8]. Thus, it was unlikely that all the Ag-positive people of all ages identified in our study acquired infection between 2015 (when American Samoa passed TAS-2) and 2016 (when TAS-3 was failed). In hindsight, early signals of ongoing transmission were evident from the population-representative serological survey of adults conducted in 2010, and further studies in 2014 which confirmed high Ag prevalence and identified Mf positive individuals within two foci of residual transmission in American Samoa [14, 16]. Ag-positive young children detected in TAS-1 and TAS-2 attended school in one of the foci identified by research studies, which could have provided early signals of focal transmission. A molecular xenomonitoring study conducted in 2011 also identified evidence of widespread low-grade infection [7], and findings were strongly geographically correlated with village-level human seroprevalence [15]. Taken together, these observations and the results of the current study indicate that, compared to current protocols and thresholds of TAS of 6–7 year old children, other surveillance strategies have the potential to detect ongoing transmission earlier and with greater geographic precision. Our findings therefore support the need for more sensitive and innovative post-MDA surveillance strategies to achieve elimination goals of the global programme.

Surveillance strategies that warrant further consideration include community-based surveys of both adults and children, school-based surveys that include a wider age range, lowering the threshold for the current TAS protocol, spatially explicit surveillance strategies, adaptive or snowball sampling (e.g. testing household and/or community members of Ag-positive children), testing for antifilarial antibodies, molecular xenomonitoring, or a combination of these strategies. We do not currently have sufficient evidence to specifically recommend any of these strategies over another, and operational research will be required to provide robust guidance for future surveillance. However, inclusion of older individuals in surveillance strategies might enable earlier detection of Ag-positive people in American Samoa and in other Polynesian countries, with highly efficient day-biting vectors and similar age-specific prevalence curves.

As countries approach the GPELF elimination targets, WHO recommends developing sustainable post-MDA surveillance strategies that are cost-efficient and can be integrated into routine surveillance activities [2, 16]. Although community-based surveys can be operationally more challenging, surveillance activities could take advantage of opportunistic and cost-effective methods of targeting community members [16, 28], such as testing high-risk occupation groups, screening at workplace clinics and antenatal clinics, or during routine health check-ups for chronic illnesses and school-based vaccination campaigns.

## Supporting information

S1 Checklist STROBE checklist for cross-sectional studies. (DOC)
